# Plasma ceramide levels are altered in low and normal birth weight men in response to short-term high-fat overfeeding

**DOI:** 10.1038/s41598-018-21419-5

**Published:** 2018-02-22

**Authors:** Amalie Ribel-Madsen, Rasmus Ribel-Madsen, Kristian Fog Nielsen, Susanne Brix, Allan A. Vaag, Charlotte Brøns

**Affiliations:** 1 Department of Biotechnology and Biomedicine, Technical University of Denmark, Kongens Lyngby, Denmark; 2 Department of Endocrinology, Diabetes and Metabolism, Copenhagen University Hospital, Copenhagen, Denmark; 3 Danish Diabetes Academy, Odense, Denmark

## Abstract

Low birth weight (LBW) individuals have an increased risk of developing insulin resistance and type 2 diabetes compared with normal birth weight (NBW) individuals. We hypothesised that LBW individuals exhibit an increased fatty acid flux into lipogenesis in non-adipose tissue with a resulting accumulation of lipotoxic lipids, including ceramides, in the blood. Therefore, we measured fasting plasma levels of 27 ceramides in 18 young, healthy, LBW men and 25 NBW controls after an isocaloric control diet and a 5-day high-fat, high-calorie diet by HPLC-HRMS. LBW men did not show elevated plasma ceramide levels after the control or high-fat, high-calorie diet. An increased fatty acid oxidation rate in these individuals during both diets may limit ceramide synthesis and thereby compensate for a likely increased fatty acid load to non-adipose tissue. Interestingly, LBW and NBW men decreased d18:0–18:1/d18:1–18:0 and d18:1–24:2/d18:2–24:1 levels and increased the d18:0–24:1a level in response to overfeeding. Plasma d18:0–24:1a and total ceramide levels were positively associated with the fasting blood glucose level and endogenous glucose production after the control diet, and the total ceramide level was in addition positively associated with hepatic insulin resistance. Further studies are needed to determine if lipotoxicity contributes to insulin resistance in LBW individuals.

## Introduction

Low birth weight (LBW) individuals have an increased risk of developing insulin resistance and type 2 diabetes later in life compared with normal birth weight (NBW) individuals^1–4^. Accordingly, we have shown that young, healthy, LBW men have higher fasting blood glucose and serum insulin levels^5^, display impaired hepatic insulin sensitivity^5^, and, in contrast to NBW men, develop impaired peripheral insulin sensitivity in response to 5-day high-fat overfeeding^6^. However, the metabolic mechanisms behind the type 2 diabetes susceptible phenotype in LBW individuals are not clear. In the present study, our primary aim was to investigate if lipotoxicity induced by ceramides could contribute to impaired hepatic insulin sensitivity in LBW men. Moreover, as we have shown that NBW men of the above mentioned study population develop impaired hepatic insulin sensitivity in response to 5-day high-fat overfeeding^6^, our secondary aim was to investigate if lipotoxicity could be part of the adverse events leading to this. Therefore, we studied fasting plasma ceramide levels, including their precursor dihydroceramides, in the LBW and NBW men of the above study population after an isocaloric control diet and after a 5-day high-fat, high-calorie diet intervention.

We have previously found that young, healthy, LBW men of another study population than the present herein display an increased whole body and adipose tissue lipolysis^7,8^. This was, however, not linked to elevated plasma non-esterified fatty acid (NEFA) or triacylglycerol levels, suggesting that an increased uptake of fatty acids from the blood into tissues and/or an increased clearance or metabolism of fatty acids in adipose tissue could be in play. Furthermore, we have demonstrated that LBW men of the present study population have a higher adipose tissue miR-483-3p level, and that manipulation of this level *in vitro* modulates adipocyte differentiation and fatty acid storage capacity^9^. Pre-adipocytes isolated from the LBW men have lower mRNA expression levels of several differentiation markers, supporting a notion of an impaired pre-adipocyte maturation in LBW men^10^. Moreover, the LBW men show an increased fatty acid oxidation rate^11^ and a higher fasting plasma acetylcarnitine level^12^, indicative of an increased fatty acid flux through beta-oxidation in mitochondria being non-matched by the acetyl-CoA flux through the tricarboxylic acid cycle. Also notably, the LBW men have higher plasma hydroxyl-/dicarboxyl-acylcarnitine levels, including 3-hydroxy-butyrylcarnitine, suggestive of an increased hepatic fatty acid oxidation rate, involving omega-oxidation in the endoplasmic reticulum, and an increased ketogenesis^12^. Omega-oxidation is a minor pathway for oxidation of fatty acids under normal physiological conditions, but an important supplementary pathway to beta-oxidation when the intracellular NEFA levels are high^13^. Taken together, we speculated that LBW individuals might have an increased fatty acid flux into non-adipose tissue, including in particular the liver, and further into lipogenesis with a resulting increased ectopic fat deposition.

Ectopic fat comprises potentially lipotoxic lipids such as long-chain acyl-CoAs, ceramides, and diacylglycerols^14^, and the extent of it has been linked to pancreatic beta-cell dysfunction and insulin resistance^15,16^. Also, individuals with type 2 diabetes have elevated plasma ceramide levels^17–19^. Importantly, obese individuals with type 2 diabetes have a higher plasma LDL-ceramide level compared with obese, insulin-sensitive individuals^19^, indicating that an increased ceramide synthesis in the liver may contribute to the development of insulin resistance^20^. Ceramides are synthesised de novo in the endoplasmic reticulum from L-serine, palmitoyl-CoA, and variable acyl-CoAs or generated in salvage pathways of other sphingolipids^21–23^. Serine palmitoyl-transferase, catalysing the condensation of L-serine and palmitoyl-CoA, is the rate-limiting enzyme of ceramide synthesis, and an increased availability of palmitic acid enhances this synthesis^24–26^. Therefore, an increased uptake of fatty acids from the blood into non-adipose tissue is expected to increase ceramide synthesis. Ceramides are potentially lipotoxic to cells in several ways^25,27^. Thus, these lipids inhibit the phosphorylation and thereby activation of Akt/protein kinase B (PKB) of the insulin signalling cascade, which is a central regulator of glucose and amino acid uptake, anabolic processes, and cell survival^28,29^. Furthermore, ceramides activate Jun and NF-KB transcription factors and may thereby enhance inflammatory responses that may interfere with insulin signalling as well^25^. A number of studies have focused on specific actions of individual ceramides, including dihydroceramides, on different cellular processes^22^. In this regard, d18:1–16:0 ceramide has been suggested to be the principal mediator of diet-induced insulin resistance^30,31^. In the present study, we hypothesised that LBW men have higher fasting plasma ceramide levels, including specific ceramide species and/or total ceramide, and that such changes are associated with their impaired insulin sensitivity.

## Results

Eighteen LBW and 25 NBW men were included in the present study. Two LBW men of the recruited participants failed to consume all the food provided during the 5-day high-fat, high-calorie (60 E% from fat, 50% extra calories) diet, and a NBW subject felt discomfort during the clamp after the control diet and therefore did not further participate in this test in the control or high-fat, high-calorie diet study part.

### Clinical characteristics

LBW and NBW men displayed several differences in glucose, lipid, and protein metabolism after the isocaloric control diet and high-fat, high-calorie diet and also differential changes in metabolism in response to the overfeeding, as published previously^5,6,11,12,32,33^. Furthermore, both birth weight groups displayed several changes in metabolism in response to the overfeeding challenge, as also published previously^6,12,33^. A selection of these findings is presented in brief below to provide background of the current findings and in Table 1 and Supplementary Table S3.

**Table 1:** Clinical characteristics of low (LBW) and normal birth weight (NBW) men following the control (C) and high-fat, high-calorie (O) diets.

	NBW (n = 25)			LBW (n = 18)			LBW vs. NBW (n = 18, n = 25)		
	C (Mean ± SD)	O (Mean ± SD)	P_NBW_	C (Mean ± SD)	O (Mean ± SD)	P_LBW_	P_C_	P_O_	P_Δ_
**Anthropometry**									
Birth weight (g)	3901 ± 207	—	—	2717 ± 268	—	—	**≤0.001**	—	—
Weight (kg)	78.4 ± 9.3	78.6 ± 9.7	n.s.	77.1 ± 11.3	77.1 ± 11.4	n.s.	n.s.	n.s.	n.s.
Height (m)	1.83 ± 0.07	—	—	1.77 ± 0.05	—	—	**≤0.05**	—	—
BMI (kg/m^2^)	23.3 ± 2.4	23.3 ± 2.5	n.s.	24.6 ± 3.8	24.6 ± 3.8	n.s.	n.s.	n.s.	n.s.
**Lipid profiling**									
P-VLDL-CHOL (mM)	0.42 ± 0.16	0.33 ± 0.16	**≤0.05**	0.49 ± 0.18	0.32 ± 0.12	**≤0.01**	n.s.	n.s.	n.s.
P-LDL-CHOL (mM)	2.51 ± 0.72	2.28 ± 0.78	**≤0.05**	2.69 ± 0.76	2.57 ± 0.80	n.s.	n.s.	n.s.	n.s.
P-HDL-CHOL (mM)	1.40 ± 0.22	1.56 ± 0.25	**≤0.01**	1.19 ± 0.23	1.38 ± 0.28	**≤0.01**	**≤0.01**	**≤0.05**	n.s.
P-CHOL (mM)	4.36 ± 0.83	4.18 ± 0.82	n.s.	4.36 ± 0.78	4.27 ± 0.79	n.s.	n.s.	n.s.	n.s.
P-TG (mM)	0.92 ± 0.35	0.73 ± 0.35	**≤0.05**	1.07 ± 0.37	0.72 ± 0.24	**≤0.01**	n.s.	n.s.	n.s.
**Clamp**									
*Basal*									
B-Glucose (mM)	4.59 ± 0.47	5.05 ± 0.40	**≤0.001**	4.97 ± 0.48	5.18 ± 0.34	**≤0.05**	**≤0.01**	n.s.	n.s.
S-Insulin (pM)	30.2 ± 14.7	43.4 ± 29.2	**≤0.05**	41.7 ± 14.6	44.7 ± 21.9	n.s.	**≤0.01**	n.s.	n.s.
P-NEFA (µM)	334 ± 136	205 ± 82	**≤0.001**	406 ± 200	188 ± 91	**≤0.001**	n.s.	n.s.	n.s.
HGP (mg/kg·FFM/min)	2.21 ± 0.48	2.85 ± 0.99	**≤0.01**	2.40 ± 0.50	2.48 ± 0.50	n.s.	n.s.	n.s.	**≤0.05**
Hepatic IR (mg/kg·FFM/min ·pM)	68.7 ± 34.1	113.7 ± 61.5	**≤0.001**	102.3 ± 50.8	108.7 ± 55.5	n.s.	**≤0.05**	n.s.	**≤0.05**
GOX (mg/kg·FFM/min)	2.34 ± 0.76	2.43 ± 0.71	n.s.	1.95 ± 0.78	2.20 ± 0.56	n.s.	n.s.	n.s.	n.s.
FOX (mg/kg·FFM/min)	1.00 ± 0.38	1.02 ± 0.33	n.s.	1.11 ± 0.53	1.17 ± 0.33	n.s.	n.s.	n.s.	n.s.
*Insulin-stimulated*									
P-NEFA (µM)	9.29 ± 4.39	12.42 ± 6.43	**≤0.01**	9.56 ± 5.03	14.39 ± 7.76	**≤0.01**	n.s.	n.s.	n.s.
M-value (mg/kg·FFM/min)	13.73 ± 2.32	13.29 ± 3.32	n.s.	13.47 ± 3.14	11.89 ± 3.57	**≤0.05**	n.s.	n.s.	n.s.
GOX (mg/kg·FFM/min)	5.18 ± 0.82	5.04 ± 0.98	n.s.	4.95 ± 0.92	4.78 ± 0.82	n.s.	n.s.	n.s.	n.s.
FOX (mg/kg·FFM/min)	0.01 ± 0.25	0.17 ± 0.33	n.s.	0.13 ± 0.46	0.37 ± 0.35	**≤0.05**	n.s.	**≤0.05**	n.s.
**IVGTT**									
FPIR (pM)	1894 ± 1431	2604 ± 1793	**≤0.001**	2135 ± 1034	2750 ± 1509	**≤0.01**	n.s.	n.s.	n.s.
Hepatic DI	0.38 ± 0.63	0.25 ± 0.21	n.s.	0.21 ± 0.11	0.24 ± 0.13	n.s.	n.s.	n.s.	n.s.
Peripheral DI	0.29 ± 0.19	0.35 ± 0.20	**≤0.05**	0.33 ± 0.13	0.32 ± 0.17	n.s.	n.s.	n.s.	n.s.

Data are presented as mean values ± standard deviations (SD). P-values are presented unadjusted for multiple comparisons, and P-values ≤ 0.05 are considered statistically significant. P_NBW_ and P_LBW_: O vs. C diet within each birth weight group, P_C_ and P_O_: LBW vs. NBW men within each diet, P_Δ_: LBW vs. NBW men on response values. P-values ≤ 0.05 are marked in bold. n.s.: Not significant. Other abbreviations: B: Blood, CHOL: Cholesterol, DI: Disposition index, FFM: Fat free mass, FOX: Fatty acid oxidation, FPIR: First phase insulin response, GOX: Glucose oxidation, HGP: Hepatic glucose production, IR: Insulin resistance, P: Plasma, S: Serum, TG: Triacylglycerol.

LBW men had higher fasting blood glucose (P ≤ 0.01) and serum insulin (P ≤ 0.01) levels after the control diet compared with NBW men^5^ (Table 1). Also, both LBW and NBW men increased the fasting blood glucose level (P ≤ 0.05 and P ≤ 0.001, respectively) in response to overfeeding, and NBW men additionally increased the fasting serum insulin level (P ≤ 0.05) due to this challenge^6^. LBW men had a higher hepatic insulin resistance index (P ≤ 0.05) after the control diet compared with NBW men^5^, and NBW men, but not LBW men, showed an increase in the hepatic glucose production (P ≤ 0.01) and in the hepatic insulin resistance index (P ≤ 0.001) in response to overfeeding^6^ (Table 1). LBW and NBW men did not show a different insulin-stimulated glucose infusion rate (M-value) after the control diet^5^ (Table 1). LBW men, however, in contrast to NBW men, decreased this rate (P ≤ 0.05) in reaction to overfeeding^6^. In terms of plasma lipid profiles, LBW men had a lower fasting plasma HDL-cholesterol level (P ≤ 0.01 and P ≤ 0.05, respectively) after both the control and high-fat, high-calorie diets compared with NBW men^6^ (Table 1). Furthermore, both LBW and NBW men decreased fasting plasma total NEFA (both P ≤ 0.001), VLDL-cholesterol (P ≤ 0.01 and P ≤ 0.05, respectively), and total triacylglycerol (P ≤ 0.01 and P ≤ 0.05, respectively) levels and increased the HDL-cholesterol level (both P ≤ 0.01) in response to overfeeding^6^ (Table 1). LBW and NBW men did not show differences in basal glucose or fatty acid oxidation rates after the control or high-fat, high-calorie diet when evaluated from the indirect calorimetry in connection with the clamp, and they also did not change these rates in response to overfeeding^6^ (Table 1). However, LBW men had higher fatty acid oxidation rates (P = 0.05/0.07 and P = 0.10, respectively) and a lower glucose oxidation rate (P = 0.05 and P = 0.06, respectively) at night or during sleep during both the control and high-fat, high-calorie diets, compared with NBW men, when studied during the 24-hour indirect calorimetry^11,32^ (Supplementary Table S3). Also, both LBW and NBW men increased the fatty acid oxidation rate in all four reported time intervals during the 24-hour indirect calorimetry in reaction to overfeeding (both P < 0.0001 for the 24-hour period)^12^. Moreover, both LBW and NBW men increased the total energy expenditure in all four time intervals due to this challenge (P = 0.0008 and P = 0.0005, respectively, for the 24-hour period)^12^ (Supplementary Table S3).

### Ceramide levels and their relation to other lipid levels and physiological measures

LBW and NBW men did not show any differences in fasting plasma ceramide levels after the control or high-fat, high-calorie diet intervention, but both birth weight groups changed plasma levels of several ceramide species in response to overfeeding (Table 2

**Table 2:** Plasma ceramide levels in low (LBW) and normal birth weight (NBW) men following the control (C) and high-fat, high-calorie (O) diets.

	**NBW** (n = 25)			**LBW** (n = 18)			**LBW vs. NBW** (n = 18, n = 25)		
(µM)	**C** (Mean, CI)	**O** (Mean, CI)	**P**_**NBW**_ **Q**_**NBW**_	**C** (Mean, CI)	**O** (Mean, CI)	**P**_**LBW**_ **Q**_**LBW**_	**P**_**C**_ **Q**_**C**_	**P**_**O**_ **Q**_**O**_	**P**_**Δ**_ **Q**_**Δ**_
**Lipid profiling**									
**Ceramides**									
d18:0-16:0	0.39 (0.25, 0.54) (n = 2)	0.39 (0.38, 0.41) (n = 8)	— (n = 1)	— — (n = 0)	0.39 (0.38, 0.39) (n = 9)	— (n = 0)	—	—	—
d18:0-23:0	0.31 (0.29, 0.33) (n = 5)	0.30 (0.11, 0.50) (n = 2)	— (n = 0)	0.30 (0.24, 0.36) (n = 3)	0.34 (0.24, 0.45) (n = 3)	— (n = 0)	—	—	—
d18:1-14:0	0.42 (0.42, 0.42) (n = 3)	0.41 (0.40, 0.42) (n = 6)	— (n = 1)	0.42 (0.37, 0.48) (n = 3)	0.41 (0.40, 0.42) (n = 7)	— (n = 3)	—	—	—
d18:0-16:1/ d18:1-16:0	0.68 (0.65, 0.72) (n = 25)	0.64 (0.60, 0.67) (n = 25)	**0.0093** **0.0349** (n = 25)	0.68 (0.64, 0.72) (n = 18)	0.64 (0.61, 0.68) (n = 18)	0.0553 (n = 18)	0.9317	0.7598	0.7528
d18:0-18:1/ d18:1-18:0	0.54 (0.51, 0.58) (n = 25)	0.47 (0.45, 0.50) (n = 23)	**<0.0001** **0.0005** (n = 23)	0.55 (0.52, 0.59) (n = 18)	0.49 (0.46, 0.52) (n = 18)	**0.0004** **0.0030** (n = 18)	0.4717	0.4904	0.6504
d18:0-20:1/ d18:1-20:0	0.56 (0.52, 0.60) (n = 25)	0.53 (0.49, 0.57) (n = 25)	0.0511 (n = 25)	0.56 (0.51, 0.60) (n = 18)	0.55 (0.50, 0.59) (n = 18)	0.5866 (n = 18)	0.3612	0.5177	0.4032
d18:0-21:1/ d18:1-21:0	0.39 (0.37, 0.41) (n = 24)	0.39 (0.37, 0.41) (n = 22)	0.9066 (n = 21)	0.40 (0.37, 0.43) (n = 16)	0.40 (0.37, 0.42) (n = 18)	0.7817 (n = 16)	0.4361	0.4592	0.8432
d18:0-22:1	— — (n = 0)	0.31 (0.29, 0.32) (n = 7)	— (n = 0)	— — (n = 0)	0.31 (0.28, 0.34) (n = 3)	— (n = 0)	—	—	—
d18:1-22:0	0.93 (0.86, 1.01) (n = 25)	0.96 (0.89, 1.04) (n = 25)	0.3741 (n = 25)	0.93 (0.85, 1.01) (n = 18)	0.98 (0.91, 1.06) (n = 18)	0.1514 (n = 18)	0.8353	0.6164	0.5904
d18:0-23:1/ d18:1-23:0	1.03 (0.93, 1.13) (n = 25)	1.11 (1.02, 1.20) (n = 25)	0.1032 (n = 25)	1.05 (0.94, 1.15) (n = 18)	1.12 (1.02, 1.23) (n = 18)	0.1684 (n = 18)	0.7634	0.8162	0.9146
d18:0-24:1a	0.34 (0.32, 0.37) (n = 24)	0.39 (0.35, 0.42) (n = 24)	**0.0147** **0.0368** (n = 23)	0.35 (0.32, 0.38) (n = 18)	0.40 (0.37, 0.43) (n = 18)	**0.0062** **0.0310** (n = 18)	0.7144	0.4813	0.7271
d18:0-24:1b	0.27 (0.26, 0.28) (n = 7)	0.27 (0.22, 0.33)(n = 2)	— (n = 2)	0.27 (0.25, 0.28) (n = 6)	0.26 (0.14, 0.37) (n = 2)	— (n = 1)	—	—	—
d18:1-24:0	2.46 (2.24, 2.69) (n = 25)	2.50 (2.23, 2.76) (n = 25)	0.7670 (n = 25)	2.36 (2.09, 2.63) (n = 18)	2.60 (2.37, 2.83) (n = 18)	0.1415 (n = 18)	0.8736	0.5577	0.5826
d18:0-25:1a	0.23 (0.21, 0.25) (n = 3)	0.24 (0.22, 0.27) (n = 7)	— (n = 3)	0.24 (0.14, 0.34)(n = 2)	0.25 (0.10, 0.40) (n = 2)	— (n = 1)	—	—	—
d18:0-25:1b/ d18:1-25:0	0.83 (0.76, 0.90) (n = 23)	0.84 (0.75, 0.93) (n = 21)	0.4492 (n = 20)	0.76 (0.65, 0.86) (n = 17)	0.81 (0.72, 0.89) (n = 14)	0.3151 (n = 14)	0.1708	0.4947	0.9980
d18:0-26:1/ d18:1-26:0	0.35 (0.32, 0.38) (n = 25)	0.35 (0.32, 0.38) (n = 24)	0.9815 (n = 24)	0.33 (0.30, 0.36) (n = 18)	0.35 (0.32, 0.38) (n = 18)	0.1540 (n = 18)	0.4142	0.8835	0.2877
d18:0-16:2/ d18:1-16:1/ d18:2-16:0	0.40 (0.39, 0.41) (n = 12)	0.40 (0.37, 0.44) (n = 3)	— (n = 3)	0.40 (0.39, 0.40) (n = 8)	0.40 (0.38, 0.42) (n = 3)	— (n = 2)	—	—	—
d18:0-18:2/ d18:1-18:1/ d18:2-18:0	0.38 (0.37, 0.40) (n = 10)	0.37 — (n = 1)	— (n = 1)	0.37 (0.35, 0.39) (n = 4)	0.37 (0.36, 0.37) (n = 3)	— (n = 1)	—	—	—
d18:0-20:2/ d18:1-20:1/ d18:2-20:0	0.35 (0.33, 0.37) (n = 6)	0.35 (0.33, 0.37) (n = 2)	— (n = 2)	0.36 (0.30, 0.41) (n = 2)	0.34 — (n = 1)	— (n = 0)	—	—	—
d18:1-22:1/ d18:2-22:0	0.61 (0.55, 0.67) (n = 25)	0.55 (0.49, 0.61) (n = 24)	**0.0072** **0.0349** (n = 24)	0.60 (0.53, 0.66) (n = 18)	0.55 (0.50, 0.60) (n = 18)	0.0850 (n = 18)	0.7229	0.9095	0.6341
d18:1-23:1/ d18:2-23:0	0.43 (0.39, 0.47) (n = 24)	0.39 (0.36, 0.42) (n = 25)	**0.0129** **0.0368** (n = 24)	0.41 (0.37, 0.44) (n = 16)	0.40 (0.37, 0.43) (n = 16)	0.8063 (n = 14)	0.3331	0.3484	0.4277
d18:1-24:1	1.80 (1.61, 1.98) (n = 25)	1.67 (1.49, 1.85) (n = 25)	0.1996 (n = 25)	1.74 (1.54, 1.95) (n = 18)	1.68 (1.52, 1.85) (n = 18)	0.4831 (n = 18)	0.7598	0.5826	0.6266
d18:0-25:2/ d18:1-25:1/ d18:2-25:0	0.42 (0.35, 0.48) (n = 25)	0.46 (0.41, 0.51) (n = 25)	0.1854 (n = 25)	0.42 (0.33, 0.50) (n = 16)	0.44 (0.38, 0.51) (n = 18)	0.0649 (n = 16)	0.8421	0.6268	0.9093
d18:0-26:2/ d18:1-26:1/ d18:2-26:0	0.25 (0.23, 0.28) (n = 15)	0.23 (0.22, 0.24) (n = 20)	— (n = 12)	0.23 (0.21, 0.25) (n = 12)	0.24 (0.23, 0.26) (n = 13)	— (n = 10)	—	—	—
d18:1-24:2/ d18:2-24:1	0.87 (0.77, 0.97) (n = 25)	0.66 (0.55, 0.76) (n = 24)	**0.0003** **0.0023** (n = 24)	0.84 (0.73, 0.95) (n = 18)	0.62 (0.52, 0.73) (n = 18)	**0.0002** **0.0030** (n = 18)	0.4659	0.8498	0.9681
d18:0-26:3/ d18:1-26:2	0.27 (0.24, 0.31) (n = 20)	0.31 (0.17, 0.45) (n = 5)	— (n = 5)	0.29 (0.25, 0.32) (n = 13)	0.37 (0.32, 0.42) (n = 7)	— (n = 4)	—	—	—
d18:0-26:4b/ d18:1-26:3/ d18:2-26:2	0.15 (0.13, 0.17) (n = 5)	0.16 (0.00, 0.32) (n = 2)	— (n = 0)	0.16 (0.13, 0.19) (n = 3)	0.13 (0.08, 0.18) (n = 3)	— (n = 1)	—	—	—
**Total levels**									
d18:0—	0.53 (0.39, 0.66) (n = 25)	0.70 (0.53, 0.87) (n = 25)	0.0778 (n = 25)	0.52 (0.41, 0.62) (n = 18)	0.76 (0.56, 0.97) (n = 18)	**0.0197** (n = 18)	0.9244	0.6176	0.6015
All	13.21 (11.93, 14.49) (n = 25)	12.39 (11.22, 13.55) (n = 25)	0.1549 (n = 25)	12.71 (11.30, 14.12) (n = 18)	12.82 (11.61, 14.02) (n = 18)	0.8709 (n = 18)	0.7785	0.4569	0.2853

Data are presented as mean values with 95% confidence intervals (CI). P-values ≤ 0.05 are presented together with Q-values, and P-values ≤ 0.05 with corresponding Q-values ≤ 0.05 are considered statistically significant. P-values were not calculated, if more than 25% of the values within one of the two groups to be compared were missing. P_NBW_ and P_LBW_: O vs. C diet within each birth weight group, P_C_ and P_O_: LBW vs. NBW men within each diet, P_Δ_: LBW vs. NBW men on response values. P-values ≤ 0.05 and Q-values ≤ 0.05 are marked in bold. Total d18:0− levels: d18:0–16:0, d18:0–23:0, d18:0–22:1, d18:0–24:1a, d18:0–24:1b, and d18:0–25:1a, Total ceramide levels: All 27 ceramide species (individual or pools).

). Thus, both LBW and NBW men significantly decreased d18:0–18:1/d18:1–18:0 (P = 0.0004 and P < 0.0001, respectively) and d18:1–24:2/d18:2–24:1 (P = 0.0002 and P = 0.0003, respectively) levels and increased the d18:0–24:1a level (P = 0.0062 and P = 0.0147, respectively) in response to overfeeding. NBW men furthermore decreased d18:0–16:1/d18:1–16:0 (P = 0.0093), d18:1–22:1/d18:2–22:0 (P = 0.0072), and d18:1–23:1/d18:2–23:0 (P = 0.0129) levels and showed a tendency to decrease the d18:0–20:1/d18:1–20:0 level (P = 0.0511) in response to overfeeding, while LBW men showed a tendency to decrease d18:0–16:1/d18:1–16:0 (P = 0.0553) and d18:1–22:1/d18:2–22:0 (P = 0.0850) levels and increase the d18:0–25:2/d18:1–25:1/d18:2–25:0 level (P = 0.0649) due to this challenge.

**Figure 1:**
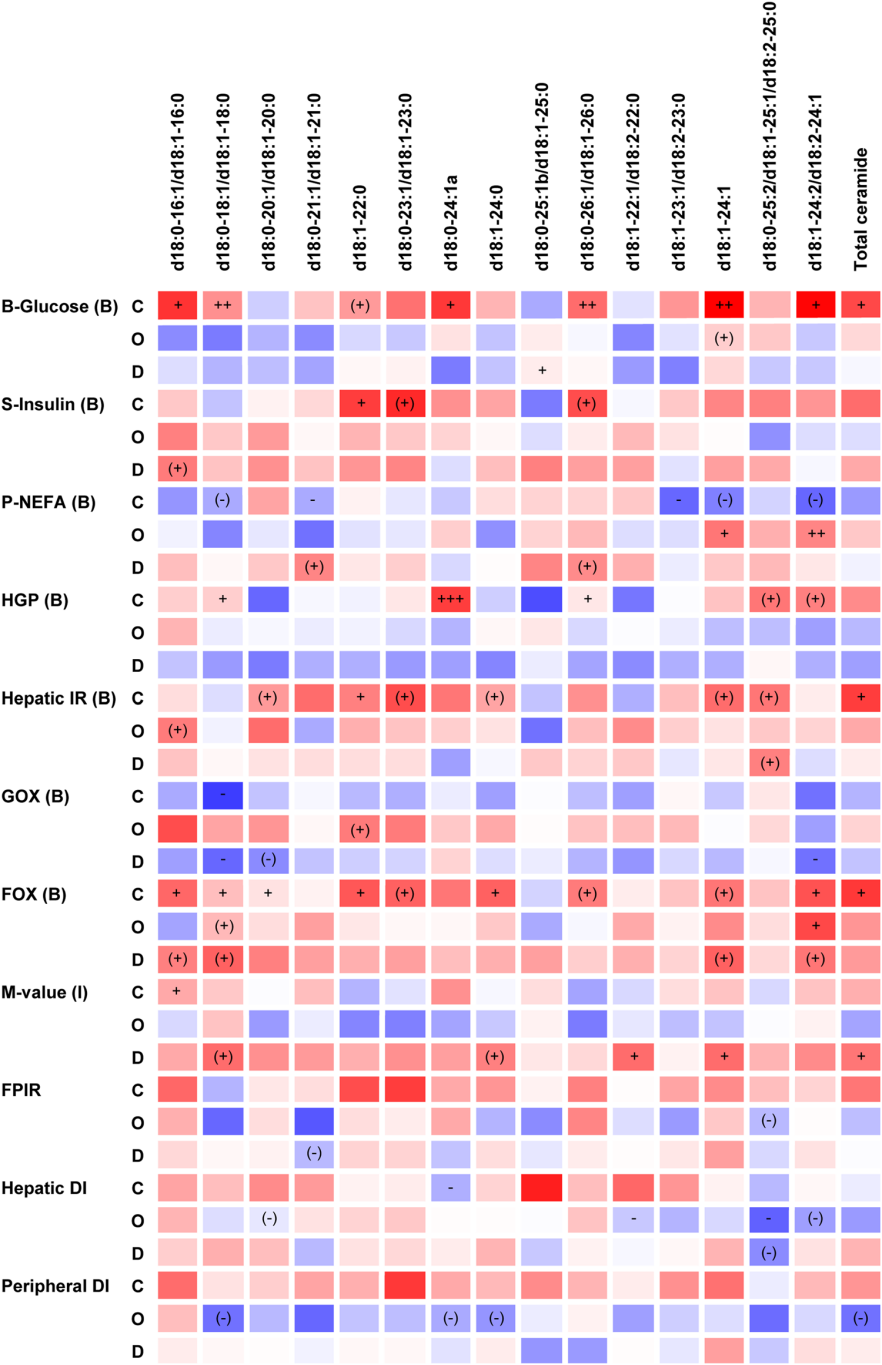
Heat-map of associations between plasma ceramide levels and physiological measures following the control (C) and high-fat, high-calorie (O) diets and between response values (D). Data are presented as r-values (red or blue colour variations for positive or negative values, respectively) and P-values (+/−: P ≤ 0.05, ++/− −: P ≤ 0.01, +++/−−−: P ≤ 0.001, (+)/(−): P ≤ 0.1 for positive or negative associations, respectively). r-values are in the range −0.4 to 0.4. P-values are presented unadjusted for multiple comparisons, and P-values ≤ 0.05 are considered statistically significant. Regression analyses were performed on the pooled data set of LBW and NBW men and were adjusted for age, BMI, and birth weight group. B or I indicated in parentheses after clamp measures specify basal or insulin-stimulated state, respectively. Other abbreviations: See Table 1.

Plasma levels of many of the detected ceramides as well as of total ceramide were positively associated with fasting plasma VLDL-cholesterol, LDL-cholesterol, total cholesterol, and total triacylglycerol levels after both the control and high-fat, high-calorie diets (Supplementary Table S4). Furthermore, d18:0–16:1/d18:1–16:0 (P = 0.0298), d18:0–18:1/d18:1–18:0 (P = 0.0076), d18:1–22:0 (P = 0.0819), d18:0–24:1a (P = 0.0144), d18:0–26:1/d18:1–26:0 (P = 0.0054), d18:1–24:1 (P = 0.0049), d18:1–24:2/d18:2–24:1 (P = 0.0109), and total ceramide (P = 0.0349) levels were or tended to be positively associated with the fasting blood glucose level after the control diet (Fig. 1). Among these ceramides, d18:0–18:1/d18:1–18:0 (P = 0.0192), d18:0–24:1a (P = 0.0010), d18:0–26:1/d18:1–26:0 (P = 0.0362), and d18:1–24:2/d18:2–24:1 (P = 0.0879) levels were or tended to be positively associated with the hepatic glucose production, and d18:1–22:0 (P = 0.0205), d18:1–24:1 (P = 0.0561), and total ceramide (P = 0.0252) levels were or tended to be positively associated with the hepatic insulin resistance index (Fig. 1). Also, d18:0–16:1/d18:1–16:0 (P = 0.0263), d18:0–18:1/d18:1–18:0 (P = 0.0214), d18:1–22:0 (P = 0.0279), d18:0–26:1/d18:1–26:0 (P = 0.0912), d18:1–24:1 (P = 0.0927), d18:1–24:2/d18:2–24:1 (P = 0.0376), and total ceramide (P = 0.0326) levels were or tended to be positively associated with the fatty acid oxidation rate determined in connection with the clamp examination after the control diet (Fig. 1).

An increase in the d18:0–25:2/d18:1–25:1/d18:2–25:0 level (P = 0.0578) in response to overfeeding tended to associate with an increase in the hepatic insulin resistance index (Fig. 1). Furthermore, decreases in d18:0–18:1/d18:1–18:0 (P = 0.0607), d18:1–24:0 (P = 0.0793), d18:1–22:1/d18:2–22:0 (P = 0.0487), d18:1–24:1 (P = 0.0437), and total ceramide (P = 0.0336) levels due to overfeeding were or tended to be associated with a decrease in the insulin-stimulated glucose uptake rate (M-value) (Fig. 1).

## Discussion

In contrast to expected, LBW men did not show altered fasting plasma ceramide levels after the control or high-fat, high-calorie diet intervention compared with NBW men. The increased fatty acid oxidation rate in the LBW men during both diets^11,32^ may limit the amount of fatty acid substrates available for de novo ceramide synthesis and thereby compensate for a possible increased fatty acid load to non-adipose tissue in these individuals. Both LBW and NBW men decreased plasma levels of several ceramide species in response to high-fat overfeeding, all of these d18:0-/d18:1- or d18:1-/d18:2- levels with the d18:1- species expected to be the dominant isomer in the pools, and increased the d18:0–24:1a level. Our findings of decreases in plasma levels of several ceramides in response to overfeeding are consistent with decreases in fasting plasma levels of several other lipid classes in the LBW and NBW men due to this challenge, including total NEFA, VLDL-cholesterol, total triacylglycerol, and total acylcarnitine levels^6,12^. The decreases in fasting plasma levels of several lipids, including a number of ceramide species, in the LBW and NBW men in response to overfeeding could very like be a result of their markedly increases in fatty acid oxidation rates and total energy expenditures due to this challenge^12^.

Several studies have reported altered plasma and/or tissue ceramide levels in mice exposed to high-fat feeding^34–37^, and a recent study has described changes in serum ceramide levels in human subjects exposed to high-fat overfeeding^38^. Thus, sedentary women and men decrease fasting serum d18:0–18:0, d18:1–18:0, and d18:1–24:1 levels and increase d18:0–22:0, d18:0–24:0, d18:1–22:0, d18:1–24:0, and total d18:1- ceramide levels in response to 28-day high-fat overfeeding (45 E% from fat, 1,250 extra kcal)^38^. Furthermore, these individuals increase fasting serum HDL-cholesterol and total cholesterol levels in response to overfeeding, while they do not change LDL-cholesterol, total NEFA, and total triacylglycerol levels^38^. Important differences between this and the present overfeeding study are the duration and fat contents of the high-fat, high-calorie diet interventions. The increases in fatty acid oxidation rates and total energy expenditures in the LBW and NBW men in response to the 5-day high-fat overfeeding could be a transient, compensatory mechanism to prevent an accumulation of lipids in non-adipose tissue and may not persist for long-term high-fat overfeeding exposures. This hypothesis, however, requires further investigations. Also, both genders are examined in the reported 28-day overfeeding study and these individuals are somewhat older (21–65 years of age) (37 ± 2 years of age)^38^ than the presently examined men (23–27 years of age) (24 ± 1 years of age). Among the studies performed in mice, several different experimental setups in regard to the high-fat diet intervention have been used^34–37^. Thus, wild-type mice fed a 16-week high-fat (60 E% from fat) diet have higher plasma levels of all measured ceramides, including seven d18:1- species, compared with mice fed a low-fat (10 E% from fat) diet, and also higher plasma total ceramide and adipose tissue total ceramide levels^34^. In addition, mice fed an 8-week high-fat (60 E% from fat) diet have higher liver total ceramide, including seven d18:1- species, and total diacylglycerol contents compared with mice fed a standard chow^35^. Plasma ceramide levels were not measured in these mice^35^. Interestingly, adiponectin administration to the mice fed the 8-week high-fat diet, and in addition to leptin deficient (ob/ob) mice, rapidly normalises liver ceramide, but not diacylglycerol, contents, regardless of ceramide species (d18:0− or d18:1−)^35^. Also, adiponectin administration to the ob/ob mice results in a reduction of the hepatic glucose production and an improvement in hepatic, but not skeletal muscle, insulin sensitivity^35^. Notably, adiponectin exerted these effects through lowering of the liver ceramide content via receptor-mediated enhancement of ceramidase activities^35^. Previously, we have found that both LBW and NBW men of the present study population increase the fasting plasma adiponectin level in response to overfeeding^6^. This, besides to their increases in fatty acid oxidation rates, may prevent an accumulation of ceramides in the liver. Furthermore, the LBW and NBW men examined herein increase the fasting serum fibroblast growth factor 21 (FGF-21) level in response to the overfeeding challenge, apparently due to an increased FGF-21 secretion from the liver^39^. FGF-21 administration to mice fed a high-fat (40 E% from fat) diet, and in addition to ob/ob mice, increases fatty acid oxidation rates and total energy expenditures and reduces hepatosteatosis^40^. A recent study has shown that FGF-21 stimulates adiponectin secretion and reduces serum ceramide levels in mice fed a high-fat (60 E% from fat) diet^41^. Also, adiponectin-knockout mice are refractory to FGF-21 effects, including lowering of ceramide levels^41^. Therefore, it was concluded that FGF-21 depends on adiponectin to exert its insulin-sensitising effects^41^.

We furthermore showed that higher plasma levels of several ceramides as well as of total ceramide were associated with a higher fasting blood glucose level and a higher degree of hepatic insulin resistance after the control diet. These findings are consistent with the observed link between liver ceramide contents and hepatic insulin sensitivity in the mice models used to investigate adiponectin effects^35^. A recent study has moreover shown that cultured rat hepatocytes exposed to high concentrations of palmitic acid have higher intracellular ceramide concentrations and are less responsive to insulin^26^. The latter was caused by an inhibition of the insulin-stimulated phosphorylation of Akt/PKB and glycogen synthase kinase^26^. The NBW men develop impaired hepatic insulin sensitivity and increase the hepatic glucose production in response to high-fat overfeeding, while the LBW men show impaired hepatic insulin sensitivity already after the control diet and do not reduce this sensitivity further in response to the overfeeding challenge^5,6^. Our findings of decreases in plasma levels of several ceramides in response to overfeeding in the NBW men do not support a possible role of ceramides in promoting hepatic insulin resistance in these individuals. Nevertheless, it is remarkable that some d18:0- species, or dihydroceramides, were only or predominantly detected in plasma from the LBW and NBW men after the high-fat, high-calorie diet (Table 2). Furthermore, the LBW and NBW men solely increased the d18:0–24:1a level in response to overfeeding. A higher d18:0–24:1a level was strongly significantly associated with an increased hepatic glucose production after the control diet. Interestingly, a newly study has reported that individuals who progress to type 2 diabetes have elevated plasma levels of specific long-chain fatty acid-containing d18:0- species several years before disease onset^42^. Moreover, we found that decreases in plasma levels of several ceramides and total ceramide in response to overfeeding were associated with a decrease in peripheral insulin sensitivity. The LBW men, but not NBW men, develop impaired peripheral insulin sensitivity in response to the high-fat overfeeding challenge^6^. Several studies have reported negative correlations between skeletal muscle total ceramide contents and insulin sensitivity^43–45^. One of these studies additionally reports a positive correlation between skeletal muscle levels of some ceramide species and insulin sensitivity in older individuals^45^. Interestingly, it has been shown that circulating LDL-ceramide in mice specifically targets skeletal muscle and induces insulin resistance^19^, emphasising the important role of ceramide metabolism in the liver. Here, we demonstrated that higher plasma levels of most of the detected ceramide species as well as of total ceramide were strongly significantly associated with higher fasting plasma VLDL- and LDL-cholesterol levels after both the control and high-fat, high-calorie diets. However, it is unknown to what extent circulating VLDL- or LDL-ceramides formed in the liver versus ceramides synthesised in skeletal muscle interfere with insulin signalling in this tissue.

Our study is the first to investigate plasma ceramide profiles in LBW men at risk of developing type 2 diabetes, compared with NBW men, and among a few studies to examine plasma ceramide profiles in human subjects exposed to short-term high-fat overfeeding. The ceramide profiles reported herein are moreover very detailed and related to measures of both hepatic and peripheral insulin sensitivity. In conclusion, LBW men did not show altered fasting plasma ceramide levels after the control or high-fat, high-calorie diet intervention compared with NBW men. We suggest that the increased fatty acid oxidation rate in the LBW men during both diets^11,32^ may limit the amount of fatty acid substrates available for lipogenesis, including de novo ceramide synthesis, and thereby may compensate for a likely increased fatty acid load to non-adipose tissue in these individuals. Both LBW and NBW men decreased plasma levels of several ceramide species in response to overfeeding. This could very likely be a result of their increases in fatty acid oxidation rates and total energy expenditures due to the overfeeding challenge^12^, potentially evoked by their increases in fasting serum FGF-21^39^ and plasma adiponectin^6^ levels. Alternatively, it might be a result of a FGF-21 and adiponectin mediated activation of ceramidases in the liver with a following increase in the ceramide degradation. A higher plasma total ceramide level was associated with a higher degree of hepatic insulin resistance after the control diet. Further studies are needed to determine if an accumulation of potentially lipotoxic lipids in tissue and plasma is part of the adverse metabolic events leading to insulin resistance in LBW individuals. Also, additional investigations are required to determine possible specific roles of individual ceramides, including dihydroceramides, in interfering with insulin signalling in the liver and skeletal muscle.

## Methods

Ceramide analyses were performed on plasma samples from LBW and NBW men subjected to dietary interventions and physiological tests prior to the present study, as described in short below and in earlier publications^5,6,11,12,32^.

### Study population

Forty-six young (23–27 years of age), healthy men were recruited from the Danish National Birth Registry according to birth weight. Among these, 20 men had a LBW (2717 ± 268 g) (≤10th percentile) and 26 men a NBW (3901 ± 207 g) (50–90th percentile). All men were born at term (39–41 weeks of gestation) in Copenhagen from 1979–1980. Also, all men were non-obese (BMI <30 kg/m^2^), did not perform strenuous physical activity >10 h/week, and did not have a family history of diabetes in two generations.

### Study design

#### Diet interventions

All men were, in a randomised crossover study setup, standardised with regard to diet and physical activity and following subjected to a 3-day control diet and a 5-day high-fat, high-calorie diet intervention separated by a 6–8 week wash out-period. The control diet was prepared to reflect a habitual, weight-maintaining diet (2,819 ± 238 kcal/day) with 15, 50, and 35 E% from protein, carbohydrate, and fat, respectively, and the high-fat, high-calorie diet was composed to contain 50% extra calories (4,228 ± 334 kcal/day) with 7.5, 32.5, and 60 E% from protein, carbohydrate, and fat, respectively (Supplementary Table S1). Both diets were provided as five daily servings, and the meals were identical from day to day. Energy requirements of the individual participants were calculated from a WHO equation for men <30 years of age with a low physical activity level^46^, and dietary calculations were made in Dankost Pro (The National Food Agency, Copenhagen, Denmark).

#### Clinical examinations

Study activities were performed over 3 days with the first of these days being placed 1 or 3 days following the start of the control and high-fat, high-calorie diet intervention, respectively. Anthropometry measures were recorded on day 1. An intravenous glucose tolerance test (IVGTT) and a hyperinsulinaemic-euglycaemic clamp were carried out in the morning on day 3 to assess insulin secretion and sensitivity, as described previously^5,6^. Furthermore, indirect calorimetry was conducted throughout 24 hours from day 1–2 as well as in the basal and insulin-stimulated steady-state periods of the clamp to determine substrate oxidation rates and total energy expenditures, as also described previously^6,11,32^. Blood samples were collected prior to and during the clamp.

### Laboratory measurements

#### Ceramide analyses

Ceramide analyses were performed on EDTA-plasma samples collected following an overnight fast (10.00 PM–7.00 AM) and immediately prior to the clamp examination. These analyses included a semi-quantitative determination of 27 ceramide species (individual or pools), denoted by the sphingoid base (i.e. d18:0-, d18:1-, or d18:2-) and acyl group (e.g. −24:0) in accordance to their carbon chain length and number of double bonds (Table 2, Supplementary Table S2).

Aliquots of 200 µL plasma were spiked with 100 µL d18:1–16:0-^2^H_31_ internal standard (Avanti Polar Lipids, Alabaster, AL, USA) solution (0.4 nmol/100 µL 2:1 chloroform:methanol), and lipids were then extracted with 1.5 mL ice-cold methanol, 3.0 ice-cold mL chloroform, and 960 µL ice-cold 0.73% sodium chloride solution according to the Bligh and Dyer method^47^. After centrifugation, the lower organic phases were transferred to new glass tubes, evaporated to dryness under nitrogen gas, and re-dissolved in 200 µL chloroform. Following, the lipid extracts were applied to n-hexane washed Strata NH_2_-columns (55 µm, 500 mg, 3 mL) (Phenomenex, Torrance, CA, USA). Cholesterolesters and triacylglycerols were first eluted with 3.0 mL 100:5:5 hexane:chloroform:ethylacetate, and cholesterol, diacylglycerols, and ceramides were subsequently eluted with 6.0 mL 23:1 chloroform:methanol into separate glass tubes. Ceramide fractions were evaporated to dryness under nitrogen gas, re-dissolved in 50 µL chloroform and 100 µL iso-propanol, and transferred to HPLC vials.

For HPLC-HRMS analyses, 1.0 µL sample was injected into an UltiMate 3000 HPLC system (Dionex, Sunnyvale, CA, USA) and separated through a Kinetex C_8_-column (2.1 × 100 mm, 2.7 µm) (Phenomenex) at 60 °C by elution with a water with 20 mM formic acid (A)-1:4 iso-propanol:acetonitrile (B) gradient. An eluent flow rate of 0.4 mL/min was applied, and the gradient started at 35% B, increased linearly from 35–70% B (0.0–1.5 min) and further from 70–100% B (1.5–9.5 min), remained at 100% B (9.5–11.0 min), decreased linearly from 100–35% B (11.0–11.1 min), and finally remained at 35% B (11.1–13.0 min). Following, the effluent was introduced into a maXis HD quadrupole TOF-MS (Bruker, Bremen, Germany) equipped with an ESI ion source operated in positive ion mode with the collision energy alternating between 0 and 25 eV every 500 ms (BBCid fragmentation), thereby generating the [M+H]^+^-ion of the ceramides and [M+H–H_2_O]^+^-fragment ion as well as a fragment ion only containing the sphingoid base moiety of the ceramides. Using an aggressive dereplication approach^48^ and Bruker Target Analysis 1.3 (Bruker), ceramide species were then identified from HPLC retention times and accurate masses of the [M+H]^+^-ion (quantifier ion), [M+H–H_2_O]^+^-fragment ion (qualifier ion 1), and sphingoid base moiety fragment ion (qualifier ion 2) (for all ions with a m/z tolerance of ±10 ppm). This enables the distinction between different position isomers (e.g. d18:0–24:1 and d18:1–24:0).

A total of 90 ceramide species were searched, including ceramides with a d18:0, d18:1, or d18:2 sphingoid base in all possible combinations with a 14:0–26:0, 14:1–26:1, 14:2–26:2, 24:3–26:3, or 26:4 acyl group, listed in Supplementary Table S2 together with m/z-values of their respective [M+H]^+^-ions, [M+H–H_2_O]^+^-fragment ions, and sphingoid base moiety fragment ions. Among these, 57 ceramide species were identified in the total set of plasma samples. Some species were, however, only detected in a few samples and therefore not included in Table 2. Furthermore, the peak areas of several position isomers (e.g. d18:0–16:1 and d18:1–16:0) were combined in the HPLC chromatograms due to similar retention times of these isomers. These were therefore pooled. Also, a few ceramide species with identical molecular formulas (e.g. d18:0–24:1) were identified at two different retention times, presumably corresponding to different structural isomers of the unsaturated acyl group. These isomers are denoted a and b, respectively. Taken together, 27 ceramide species (individual or pools) were selected for quantifications and included in Table 2.

For quantifications, external standard samples, comprising a dilution row of d18:1–14:0, d18:1–24:0, d18:1–18:1, d18:1–24:1, and d18:1–16:0-^2^H_31_ standard (Avanti Polar Lipids, Alabaster, AL, USA) solutions (1:2 chloroform:iso-propanol), were analysed by HPLC-HRMS parallel to the plasma samples to determine the area-mass relationships of these species. For all standards, this was linear within the relevant concentration ranges. Ceramide peak areas in the plasma samples were adjusted for the recovery of the d18:1–16:0-^2^H_31_ internal standard (79.0 ± 3.6%) and following converted to masses from the response of their respective external standard or an estimated response. Calculations of estimated responses were based on the assumption of an equal change in response per carbon atom and per double bond in the acyl group. These changes were determined from the responses of d18:1–14:0 compared with d18:1–24:0 and d18:1–24:0 compared with d18:1–24:1, respectively. d18:0-, d18:1-, and d18:2- species with identical acyl groups were assigned the same response. Also, ceramides that were pooled were assigned the response of the d18:1- position isomer that was expected to be the dominant isomer. Total d18:0-, or dihydroceramide, levels were calculated as the sum of the d18:0–16:0, d18:0–23:0, d18:0–22:1, d18:0–24:1a, d18:0–24:1b, and d18:0–25:1a levels, and total ceramide levels were calculated as the sum of the levels of the 27 ceramide species (individual or pools) included in Table 2.

Ceramide analyses were performed at the Metabolomics Platform, Department of Biotechnology and Biomedicine, Technical University of Denmark, Kongens Lyngby, Denmark.

### Ethical approval

All study procedures were in accordance with the principles of The Declaration of Helsinki and approved by The Regional Research Ethics Committee of Copenhagen, Denmark. Furthermore, all participants were provided with written information on the study purpose and procedures and signed an informed consent prior to their participation.

### Statistical analyses

#### Ceramide levels and their relation to other lipid levels and physiological measures

Ceramide data presented in Table 2, including plasma levels of individual species within each combination of birth weight group and diet intervention and differences in these levels between the two diets (response values) within each birth weight group, were evaluated for normality by Shapiro-Wilk tests with a significance level of 0.05. Plasma levels of individual ceramide species within each diet and response values were furthermore evaluated for equality of variances between the birth weight groups by F-tests with a significance level of 0.05 as well. Statistically significant differences in plasma ceramide levels between NBW and LBW men within each diet and in response values between the birth weight groups were subsequently assessed by Student’s two-tailed, unpaired t-tests (for normally distributed values) or Wilcoxon ranked-sum tests (for non-normally distributed values). Furthermore, significant differences in plasma ceramide levels between the control and high-fat, high-calorie diets within each birth weight group were assessed by Student’s two-tailed, paired t-tests (for normally distributed values) or Wilcoxon signed-rank tests (for non-normally distributed values). P-values from Student’s t-tests and Wilcoxon tests were evaluated in context with false discovery rates (Q-values) to account for multiple testing within each diet or birth weight group. Q-values were calculated by the Benjamini and Hochberg method. P-values ≤ 0.05 with Q-values ≤ 0.05 were considered statistically significant. Data in Table 2 are presented as mean values with 95% confidence intervals together with the number of observations within each group. Moreover, P- and Q-values are indicated. Student’s t-tests and Wilcoxon tests were not performed, if more than 25% of the values within one of the two groups to be compared were missing. Total d18:0- levels were calculated as the sum of the d18:0–16:0, d18:0–23:0, d18:0–22:1, d18:0–24:1a, d18:0–24:1b, and d18:0–25:1a levels, and total ceramide levels were calculated as the sum of the levels of all 27 ceramides (individual or pools) included in Table 2.

Associations between plasma ceramide levels and other lipid levels or physiological measures within each diet and furthermore between response values, as presented in Fig. 1 and Supplementary Table S4, were assessed from linear regression analyses. These analyses were performed on the pooled data set of LBW and NBW men and were adjusted for age, BMI, and birth weight group. P-values ≤ 0.05 were considered statistically significant. Data in Fig. 1 and Supplementary Table S4 are presented as Spearman correlation coefficients (r-values) and P-values.

### Data availability

Plasma ceramide data generated in the present study are available from the corresponding author on reasonable request.

## Electronic supplementary material

Supplementary information

## References

[1] Ravelli GP, Stein ZA, Susser MW (1976). Obesity in Young Men after Famine Exposure in Utero and Early Infancy. New England Journal of Medicine.

[2] Hales CN (1991). Fetal and infant growth and impaired glucose tolerance at age 64. British Medical Journal.

[3] Barker DJP (1993). Type 2 (non-insulin-dependent) diabetes-mellitus, hypertension and hyperlipemia (syndrome-X) - relation to reduced fetal growth. Diabetologia.

[4] Harder T, Rodekamp E, Schellong K, Dudenhausen JW, Plagemann A (2007). Birth weight and subsequent risk of type 2 diabetes: A meta-analysis. American Journal of Epidemiology.

[5] Brons C (2008). Mitochondrial function in skeletal muscle is normal and unrelated to insulin action in young men born with low birth weight. Journal of Clinical Endocrinology & Metabolism.

[6] Brons C (2012). Effects of high-fat overfeeding on mitochondrial function, glucose and fat metabolism, and adipokine levels in low-birth-weight subjects. American Journal of Physiology-Endocrinology and Metabolism.

[7] Alibegovic AC (2010). Increased rate of whole body lipolysis before and after 9 days of bed rest in healthy young men born with low birth weight. American Journal of Physiology-Endocrinology and Metabolism.

[8] Hojbjerre L (2011). Increased lipolysis but diminished gene expression of lipases in subcutaneous adipose tissue of healthy young males with intrauterine growth retardation. Journal of Applied Physiology.

[9] Ferland-McCollough D (2012). Programming of adipose tissue miR-483-3p and GDF-3 expression by maternal diet in type 2 diabetes. Cell Death and Differentiation.

[10] Schultz NS (2014). Impaired leptin gene expression and release in cultured preadipocytes isolated from individuals born with low birth weight. Diabetes.

[11] Brons C (2013). Increased nocturnal fat oxidation in young healthy men with low birth weight: Results from 24-h whole-body respiratory chamber measurements. Metabolism-Clinical and Experimental.

[12] Ribel-Madsen A (2016). Plasma acylcarnitine profiling indicates increased fatty acid oxidation relative to tricarboxylic acid cycle capacity in young, healthy low birth weight men. Physiological Reports.

[13] Patsouris D, Reddy JK, Muller M, Kersten S (2006). Peroxisome proliferator-activated receptor alpha mediates the effects of high-fat diet on hepatic gene expression. Endocrinology.

[14] Boren J, Taskinen MR, Olofsson SO, Levin M (2013). Ectopic lipid storage and insulin resistance: a harmful relationship. Journal of Internal Medicine.

[15] van Herpen NA, Schrauwen-Hinderling VB (2008). Lipid accumulation in non-adipose tissue and lipotoxicity. Physiology & Behavior.

[16] Kusminski CM, Shetty S, Orci L, Unger RH, Scherer PE (2009). Diabetes and apoptosis: lipotoxicity. Apoptosis: An International Journal on Programmed Cell Death.

[17] Haus JM (2009). Plasma ceramides are elevated in obese subjects with type 2 diabetes and correlate with the severity of insulin resistance. Diabetes.

[18] Lopez X, Goldfine AB, Holland WL, Gordillo R, Scherer PE (2013). Plasma ceramides are elevated in female children and adolescents with type 2 diabetes. Journal of Pediatric Endocrinology & Metabolism.

[19] Boon J (2013). Ceramides contained in LDL are elevated in type 2 diabetes and promote inflammation and skeletal muscle insulin resistance. Diabetes.

[20] Wiesner P, Leidl K, Boettcher A, Schmitz G, Liebisch G (2009). Lipid profiling of FPLC-separated lipoprotein fractions by electrospray ionization tandem mass spectrometry. Journal of Lipid Research.

[21] Levy M, Futerman AH (2010). Mammalian ceramide synthases. IUBMB Life.

[22] Grosch S, Schiffmann S, Geisslinger G (2012). Chain length-specific properties of ceramides. Progress in Lipid Research.

[23] Barbarroja N (2015). Increased dihydroceramide/ceramide ratio mediated by defective expression of degs1 impairs adipocyte differentiation and function. Diabetes.

[24] Lightle S (2003). Elevation of ceramide in serum lipoproteins during acute phase response in humans and mice: role of serine-palmitoyl transferase. Archives of Biochemistry and Biophysics.

[25] Summers SA (2006). Ceramides in insulin resistance and lipotoxicity. Progress in Lipid Research.

[26] Konstantynowicz-Nowicka K, Harasim E, Baranowski M, Chabowski A (2015). New evidence for the role of ceramide in the development of hepatic insulin resistance. PLoS One.

[27] Chaurasia B, Summers SA (2015). Ceramides - Lipotoxic Inducers of Metabolic Disorders. Trends in Endocrinology and Metabolism.

[28] Whiteman EL, Cho H, Birnbaum MJ (2002). Role of Akt/protein kinase B in metabolism. Trends in Endocrinology and Metabolism.

[29] Stratford S, Hoehn KL, Liu F, Summers SA (2004). Regulation of insulin action by ceramide: dual mechanisms linking ceramide accumulation to the inhibition of Akt/protein kinase B. Journal of Biological Chemistry.

[30] Turpin SM (2014). Obesity-induced CerS6-dependent C16:0 ceramide production promotes weight gain and glucose intolerance. Cell Metabolism.

[31] Raichur S (2014). CerS2 haploinsufficiency inhibits beta-oxidation and confers susceptibility to diet-induced steatohepatitis and insulin resistance. Cell Metabolism.

[32] Brons C, Lilleore SK, Astrup A, Vaag A (2015). Disproportionately increased 24-h energy expenditure and fat oxidation in young men with low birth weight during a high-fat overfeeding challenge. European Journal of Nutrition.

[33] Ribel-Madsen A (2016). Plasma amino acid levels are elevated in young, healthy low birth weight men exposed to short-term high-fat overfeeding. Physiological Reports.

[34] Shah C (2008). Protection from high fat diet-induced increase in ceramide in mice lacking plasminogen activator inhibitor 1. Journal of Biological Chemistry.

[35] Holland WL (2011). Receptor-mediated activation of ceramidase activity initiates the pleiotropic actions of adiponectin. Nature Medicine.

[36] Barber MN (2012). Plasma lysophosphatidylcholine levels are reduced in obesity and type 2 diabetes. PLoS One.

[37] Eisinger K (2014). Lipidomic analysis of serum from high fat diet induced obese mice. International Journal of Molecular Sciences.

[38] Heilbronn LK (2013). The effect of short-term overfeeding on serum lipids in healthy humans. Obesity (Silver Spring, Md.).

[39] Vienberg SG (2012). Impact of short-term high-fat feeding and insulin-stimulated FGF21 levels in subjects with low birth weight and controls. European Journal of Endocrinology/European Federation of Endocrine Societies.

[40] Coskun T (2008). Fibroblast growth factor 21 corrects obesity in mice. Endocrinology.

[41] Holland WL (2013). An FGF21-adiponectin-ceramide axis controls energy expenditure and insulin action in mice. Cell Metabolism.

[42] Wigger L (2017). Plasma Dihydroceramides Are Diabetes Susceptibility Biomarker Candidates in Mice and Humans. Cell Reports.

[43] Straczkowski M (2007). Increased skeletal muscle ceramide level in men at risk of developing type 2 diabetes. Diabetologia.

[44] Ussher JR (2010). Inhibition of de novo ceramide synthesis reverses diet-induced insulin resistance and enhances whole-body oxygen consumption. Diabetes.

[45] Amati F (2011). Skeletal muscle triglycerides, diacylglycerols, and ceramides in insulin resistance: another paradox in endurance-trained athletes?. Diabetes.

[46] WHO. Human Energy Requirements. Report of a Joint FAO/WHO/UNU Expert Consultation, Rome, 17–24 October 2001. (Geneva 2001).

[47] Bligh EG, Dyer WJ (1959). A Rapid Method of Total Lipid Extraction and Purification. Canadian Journal of Biochemistry and Physiology.

[48] Klitgaard A (2014). Aggressive dereplication using UHPLC-DAD-QTOF: screening extracts for up to 3000 fungal secondary metabolites. Analytical and Bioanalytical Chemistry.

