# Supplementary material for: Plasma ceramide levels are altered in low and normal birth weight men in response to short-term high-fat overfeeding
Source: Sci Rep. 2018 Feb 22;8:3452. doi: 10.1038/s41598-018-21419-5 (PMC5823847; doi:10.1038/s41598-018-21419-5)
Supplement: Supplementary file 1 — Supplementary information [file 41598_2018_21419_MOESM1_ESM.pdf]

SUPPLEMENTARY INFORMATION

22 December 2017

TITLE

Plasma ceramide levels are altered in low and normal birth weight men in response to short-term high-fat overfeeding.

AUTHORS

Amalie Ribel-Madsen[* 1, 2], Rasmus Ribel-Madsen[2, 3], Kristian Fog Nielsen[1], Susanne Brix[1], Allan A. Vaag[2], and Charlotte Brøns[2].

AUTHOR AFFILIATIONS

[1]Department of Biotechnology and Biomedicine, Technical University of Denmark, Kongens Lyngby, Denmark, [2]Department of Endocrinology, Diabetes and Metabolism, Copenhagen University Hospital, Copenhagen, Denmark, [3]Danish Diabetes Academy, Odense, Denmark.

CORRESPONDING AUTHOR

Amalie Ribel-Madsen, Department of Biotechnology and Biomedicine, Technical University of Denmark, Søltofts Plads, DK-2800 Kongens Lyngby, Denmark, Telephone number: +45 45252744, Fax number: +45 45886307, E-mail address: amari@bio.dtu.dk.

Table S1: Protein, carbohydrate, and fat contents of the control (C) and high-fat, high-calorie (O) diets.

	**C**		**O**		**O/C**	
	**Total** (Mean)	**Per 100 g** (Mean)	**Total** (Mean)	**Per 100 g** (Mean)	**Total** (Ratio)	**Per 100 g** (Ratio)
**Energy (kJ)**						
Total	9664	698	14848	1135	1.54	1.63
**Energy (E%)**						
Protein	15	15	8	8	0.53	0.53
Carbohydrate	49	49	33	33	0.67	0.67
Fat	35	35	60	60	1.71	1.71
**Energy (g)**						
Protein	88	6.4	67.1	5.1	0.76	0.80
Carbohydrate	266.7	19.2	277.6	21.2	1.04	1.10
Fat	92.1	6.6	239	18.3	2.60	2.77
**Fat (g)**						
Saturated fatty acids	35.6	2.6	109.8	8.4	3.08	3.23
Monounsaturated fatty acids	31.5	2.3	85.4	6.5	2.71	2.83
Polyunsaturated fatty acids	8.6	0.6	28.5	2.2	3.31	3.67
n-3 fatty acids	0.9	0.1	5.8	0.4	6.44	4.00
n-6 fatty acids	7.2	0.5	21.8	1.7	3.03	3.40

Table S2: Ceramides searched in the plasma samples and mass to charge ratios (m/z-values) of their respective [M+H]$^+$-ions, [M+H–H_2O]$^+$-fragment ions, and sphingoid base moiety fragment ions. Ceramide position isomers are listed in the same rows according to the total number of double bonds in the structures with indication in parentheses of m/z-values of the [M+H]$^+$-ion (quantifier ion) and [M+H–H_2O]$^+$-fragment ion (qualifier 1 ion). d18:0-, d18:1-, and d18:2- position isomers have constant m/z-values of the sphingoid base moiety fragment ion (qualifier 2 ion) of 266.2842, 264.2686, or 262.2529 Da, respectively, and variable m/z-values of the [M+H]$^+$-ion and [M+H–H_2O]$^+$-fragment ion dependent on the acyl group in the structures. Ceramides detected in the plasma samples are marked in bold.

	**d18:0- species**	**d18:1- species**	**d18:2- species**
**Ceramides**<br><br><br>(Formula)	**Quantifier ion: [M+H]$^+$**<br>**Qualifier 1 ion: [M+H–H_2O]$^+$**<br>**Qualifier 2 ion: 266.2842 Da**<br>(Da)	**Quantifier ion: [M+H]$^+$**<br>**Qualifier 1 ion: [M+H–H_2O]$^+$**<br>**Qualifier 2 ion: 264.2686 Da**<br>(Da)	**Quantifier ion: [M+H]$^+$**<br>**Qualifier 1 ion: [M+H–H_2O]$^+$**<br>**Qualifier 2 ion: 262.2529 Da**<br>(Da)
**Double bonds**			
**0**			
$C_{32}H_{65}NO_3$	d18:0-14:0 (512.5038, 494.4932)	-	-
$C_{33}H_{67}NO_3$	d18:0-15:0 (526.5194, 508.5088)	-	-
$C_{34}H_{69}NO_3$	**d18:0-16:0** (540.5351, 522.5245)	-	-
$C_{35}H_{71}NO_3$	d18:0-17:0 (554.5507, 536.5401)	-	-
$C_{36}H_{73}NO_3$	d18:0-18:0 (568.5664, 550.5558)	-	-
$C_{37}H_{75}NO_3$	d18:0-19:0 (582.5820, 564.5714)	-	-
$C_{38}H_{77}NO_3$	d18:0-20:0 (596.5977, 578.5871)	-	-
$C_{39}H_{79}NO_3$	**d18:0-21:0** (610.6133, 592.6027)	-	-
$C_{40}H_{81}NO_3$	**d18:0-22:0** (624.6290, 606.6184)	-	-
$C_{41}H_{83}NO_3$	**d18:0-23:0** (638.6446, 620.6340)	-	-
$C_{42}H_{85}NO_3$	**d18:0-24:0** (652.6603, 634.6497)	-	-
$C_{43}H_{87}NO_3$	d18:0-25:0 (666.6759, 648.6653)	-	-
$C_{44}H_{89}NO_3$	d18:0-26:0 (680.6916, 662.6810)	-	-
**1**			
$C_{32}H_{63}NO_3$	d18:0-14:1 (510.4881, 492.4775)	**d18:1-14:0** (510.4881, 492.4775)	-
$C_{33}H_{65}NO_3$	**d18:0-15:1** (524.5038, 506.4932)	**d18:1-15:0** (524.5038, 506.4932)	-
$C_{34}H_{67}NO_3$	**d18:0-16:1** (538.5194, 520.5088)	**d18:1-16:0** (538.5194, 520.5088)	-
$C_{35}H_{69}NO_3$	**d18:0-17:1** (552.5351, 534.5245)	d18:1-17:0 (552.5351, 534.5245)	-
$C_{36}H_{71}NO_3$	**d18:0-18:1** (566.5507, 548.5401)	**d18:1-18:0** (566.5507, 548.5401)	-
$C_{37}H_{73}NO_3$	**d18:0-19:1** (580.5664, 562.5558)	d18:1-19:0 (580.5664, 562.5558)	-
$C_{38}H_{75}NO_3$	**d18:0-20:1** (594.5820, 576.5714)	**d18:1-20:0** (594.5820, 576.5714)	-
$C_{39}H_{77}NO_3$	**d18:0-21:1** (608.5977, 590.5871)	**d18:1-21:0** (608.5977, 590.5871)	-
$C_{40}H_{79}NO_3$	**d18:0-22:1** (622.6133, 604.6027)	**d18:1-22:0** (622.6133, 604.6027)	-
$C_{41}H_{81}NO_3$	**d18:0-23:1** (636.6290, 618.6184)	**d18:1-23:0** (636.6290, 618.6184)	-
$C_{42}H_{83}NO_3$	**d18:0-24:1** (650.6446, 632.6340)	**d18:1-24:0** (650.6446, 632.6340)	-

$C_{43}H_{85}NO_3$	**d18:0-25:1** (664.6603, 646.6497)	**d18:1-25:0** (664.6603, 646.6497)	-
$C_{44}H_{87}NO_3$	**d18:0-26:1** (678.6759, 660.6653)	**d18:1-26:0** (678.6759, 660.6653)	-
**2**			
$C_{32}H_{61}NO_3$	d18:0-14:2 (508.4725, 490.4619)	d18:1-14:1 (508.4725, 490.4619)	d18:2-14:0 (508.4725, 490.4619)
$C_{33}H_{63}NO_3$	d18:0-15:2 (522.4881, 504.4775)	d18:1-15:1 (522.4881, 504.4775)	d18:2-15:0 (522.4881, 504.4775)
$C_{34}H_{65}NO_3$	**d18:0-16:2** (536.5038, 518.4932)	**d18:1-16:1** (536.5038, 518.4932)	**d18:2-16:0** (536.5038, 518.4932)
$C_{35}H_{67}NO_3$	d18:0-17:2 (550.5194, 532.5088)	d18:1-17:1 (550.5194, 532.5088)	d18:2-17:0 (550.5194, 532.5088)
$C_{36}H_{69}NO_3$	**d18:0-18:2** (564.5351, 546.5245)	**d18:1-18:1** (564.5351, 546.5245)	**d18:2-18:0** (564.5351, 546.5245)
$C_{37}H_{71}NO_3$	d18:0-19:2 (578.5507, 560.5401)	d18:1-19:1 (578.5507, 560.5401)	d18:2-19:0 (578.5507, 560.5401)
$C_{38}H_{73}NO_3$	**d18:0-20:2** (592.5664, 574.5558)	**d18:1-20:1** (592.5664, 574.5558)	**d18:2-20:0** (592.5664, 574.5558)
$C_{39}H_{75}NO_3$	d18:0-21:2 (606.5820, 588.5714)	d18:1-21:1 (606.5820, 588.5714)	d18:2-21:0 (606.5820, 588.5714)
$C_{40}H_{77}NO_3$	d18:0-22:2 (620.5977, 602.5871)	**d18:1-22:1** (620.5977, 602.5871)	**d18:2-22:0** (620.5977, 602.5871)
$C_{41}H_{79}NO_3$	d18:0-23:2 (634.6133, 616.6027)	**d18:1-23:1** (634.6133, 616.6027)	**d18:2-23:0** (634.6133, 616.6027)
$C_{42}H_{81}NO_3$	d18:0-24:2 (648.6290, 630.6184)	**d18:1-24:1** (648.6290, 630.6184)	d18:2-24:0 (648.6290, 630.6184)
$C_{43}H_{83}NO_3$	**d18:0-25:2** (662.6446, 644.6340)	**d18:1-25:1** (662.6446, 644.6340)	**d18:2-25:0** (662.6446, 644.6340)
$C_{44}H_{85}NO_3$	**d18:0-26:2** (676.6603, 658.6497)	**d18:1-26:1** (676.6603, 658.6497)	**d18:2-26:0** (676.6603, 658.6497)
**3**			
$C_{42}H_{79}NO_3$	d18:0-24:3 (646.6133, 628.6027)	**d18:1-24:2** (646.6133, 628.6027)	**d18:2-24:1** (646.6133, 628.6027)
$C_{43}H_{81}NO_3$	d18:0-25:3 (660.6290, 642.6184)	**d18:1-25:2** (660.6290, 642.6184)	**d18:2-25:1** (660.6290, 642.6184)
$C_{44}H_{83}NO_3$	**d18:0-26:3** (674.6446, 656.6340)	**d18:1-26:2** (674.6446, 656.6340)	d18:2-26:1 (674.6446, 656.6340)
**4**			
$C_{44}H_{81}NO_3$	**d18:0-26:4** (672.6290, 654.6184)	**d18:1-26:3** (672.6290, 654.6184)	**d18:2-26:2** (672.6290, 654.6184)

Table S3: Glucose, fatty acid, and protein oxidation rates and total energy expenditures in low (LBW) and normal birth weight (NBW) men during the control (C) and high-fat, high-calorie (O) diets. Data are presented as mean values ± standard errors of means (SEM). P-values are presented unadjusted for multiple comparisons, and P-values ≤0.05 are considered statistically significant. P_{NBW} and P_{LBW}: O vs. C diet within each birth weight group, P_C and P_O: LBW vs. NBW men within each diet, P_Δ: LBW vs. NBW men on response values. P-values ≤0.05 are marked in bold. Day: 9 am-11 pm, Night: 11 pm-8 am, Sleep: 1 am-6 am, 24 hours: 9 am-9 am. Abbreviations: EE: Energy expenditure, FOX: Fatty acid oxidation, GOX: Glucose oxidation, POX: Protein oxidation.

	**NBW** (n = 26)			**LBW** (C: n = 20, O: n = 18)			**LBW vs. NBW** (n = 20/n = 18, n = 26)		
(kJ/min)	**C** (Mean ± SEM)	**O** (Mean ± SEM)	**P_{NBW}**	**C** (Mean ± SEM)	**O** (Mean ± SEM)	**P_{LBW}**	**P_C**	**P_O**	**P_Δ**
**Calorimetry 24 h**									
**GOX**									
Day	3.85 ± 0.17	3.50 ± 0.08	**0.0297**	3.69 ± 0.16	3.30 ± 0.14	0.0609	0.52	0.19	0.94
Night	1.97 ± 0.10	2.07 ± 0.07	0.3126	1.78 ± 0.09	1.84 ± 0.10	0.3391	0.18	0.06	0.97
Sleep	1.91 ± 0.12	1.89 ± 0.08	0.9131	1.58 ± 0.10	1.77 ± 0.11	0.0836	**0.05**	0.37	0.21
24 h	3.10 ± 0.13	2.93 ± 0.07	0.1510	2.92 ± 0.13	2.73 ± 0.09	0.2620	0.34	0.09	0.97
**FOX**									
Day	3.34 ± 0.16	4.23 ± 0.14	**<0.0001**	3.46 ± 0.14	4.52 ± 0.21	**<0.0001**	0.60	0.23	0.60
Night	2.34 ± 0.10	2.80 ± 0.10	**0.0005**	2.60 ± 0.08	3.06 ± 0.12	**0.0023**	0.07	0.10	0.93
Sleep	2.14 ± 0.14	2.72 ± 0.12	**0.0001**	2.50 ± 0.09	2.87 ± 0.13	**0.0221**	**0.05**	0.38	0.40
24 h	2.92 ± 0.12	3.63 ± 0.12	**<0.0001**	3.11 ± 0.11	3.91 ± 0.14	**<0.0001**	0.24	0.14	0.76
**POX**									
Day	1.13 ± 0.04	0.79 ± 0.03	**<0.0001**	1.08 ± 0.04	0.74 ± 0.04	**<0.0001**	0.48	0.32	0.71
Night	1.13 ± 0.04	0.79 ± 0.03	**<0.0001**	1.08 ± 0.04	0.74 ± 0.04	**<0.0001**	0.48	0.32	0.71
Sleep	1.13 ± 0.04	0.79 ± 0.03	**<0.0001**	1.08 ± 0.04	0.74 ± 0.04	**<0.0001**	0.48	0.32	0.71
24 h	1.13 ± 0.04	0.79 ± 0.03	**<0.0001**	1.08 ± 0.04	0.74 ± 0.04	**<0.0001**	0.48	0.32	0.71
**EE**									
Day	8.32 ± 0.15	8.52 ± 0.13	**0.0142**	8.24 ± 0.16	8.56 ± 0.18	**0.0021**	0.71	0.86	0.39
Night	5.43 ± 0.09	5.65 ± 0.10	**0.0001**	5.46 ± 0.11	5.66 ± 0.13	**0.0017**	0.82	0.97	0.99
Sleep	5.17 ± 0.09	5.39 ± 0.09	**0.0010**	5.16 ± 0.11	5.30 ± 0.13	**0.0009**	0.96	0.93	0.82
24 h	7.14 ± 0.12	7.36 ± 0.12	**0.0005**	7.12 ± 0.14	7.38 ± 0.15	**0.0008**	0.88	0.90	0.55

Table S4: Associations between plasma ceramide levels and other lipid levels or physiological measures following the control (C) and high-fat, high-calorie (O) diets and between response values (Δ). Data are presented as r-values (+/– for positive or negative values, respectively) and P-values (+/–: P≤0.05, + +/– –: P≤0.01, + + +/– – –: P≤0.001, (+)/(–): P≤0.1 for positive or negative associations, respectively). P-values are presented unadjusted for multiple comparisons, and P-values ≤0.05 are considered statistically significant. Regression analyses were performed on the pooled data set of LBW and NBW men and were adjusted for age, BMI, and birth weight group. Abbreviations: See Table 1.

		d18:0-16:1/d18:1-16:0	d18:0-18:1/d18:1-18:0	d18:0-20:1/d18:1-20:0	d18:0-21:1/d18:1-21:0	d18:1-22:0	d18:0-23:1/d18:1-23:0	d18:0-24:1a	d18:1-24:0	d18:0-25:1 b/d18:1-25:0	d18:0-26:1/d18:1-26:0	d18:1-22:1/d18:2-22:0	d18:1-23:1/d18:2-23:0	d18:1-24:1	d18:0-25:2/d18:1-25:1/d18:2-25:0	d18:1-24:2/d18:2-24:1	Total ceramide
**Lipid profiling**																	
P-VLDL-CHOL	**C**	+	++		+	++	++	(+)	+		(+)		+	++		+++	++
	**O**									++	(+)			++		+++	+
	**Δ**																
P-LDL-CHOL	**C**	+++				++	+	+++						++	+	+++	++
	**O**		+++	+	+	+++	+++	+	+++	+++	+++	++	+	++	+	++	+++
	**Δ**		(+)			(+)	(+)	(+)	+		+					(+)	
P-HDL-CHOL	**C**																
	**O**														(–)		
	**Δ**	+		(+)		+	+		+	++	(+)			++			+
P-CHOL	**C**	+++				+++	++	++						++	(+)	+++	++
	**O**		+++	++	++	+++	+++	+	+++	+++	+++	++	++	+++	+	+++	+++
	**Δ**	(+)	(+)			+	+	(+)	+		+			+		+	(+)
P-TG	**C**	+	+++		+	++	++		(+)		+		+	++		+++	++
	**O**		++	++	+++	++	++	(+)	++	++	+	+++	+	+++	(+)	+++	++
	**Δ**																
**Clamp**																	
Basal																	
B-Glucose	**C**	+	++			(+)		+			++			++		+	+
	**O**													(+)			
	**Δ**									+							
S-Insulin	**C**					+	(+)				(+)						
	**O**																
	**Δ**	(+)															
P-NEFA	**C**		(–)		–								–	(–)		(–)	

	**O**													+		++	
	**Δ**				(+)						(+)						
HGP	**C**		+					+++			+				(+)	(+)	
	**O**																
	**Δ**																
Hepatic IR	**C**			(+)		+	(+)		(+)					(+)	(+)		+
	**O**	(+)															
	**Δ**														(+)		
GOX	**C**		–														
	**O**					(+)											
	**Δ**		–	(–)												–	
FOX	**C**	+	+	+		+	(+)		+		(+)			(+)		+	+
	**O**		(+)													+	
	**Δ**	(+)	(+)											(+)		(+)	
Insulin-stimulated																	
M-value	**C**	+															
	**O**																
	**Δ**		(+)						(+)			+		+			+
**IVGTT**																	
FPIR	**C**																
	**O**														(–)		
	**Δ**				(–)												
Hepatic DI	**C**							–									
	**O**			(–)								–			–	(–)	
	**Δ**														(–)		
Peripheral DI	**C**																
	**O**		(–)					(–)	(–)								(–)
	**Δ**																
